# Sequential Vaccination With Heterologous *Acinetobacter baumannii* Strains Induces Broadly Reactive Antibody Responses

**DOI:** 10.3389/fimmu.2021.705533

**Published:** 2021-07-30

**Authors:** Gathoni Kamuyu, Yat Suen Cheng, Sam Willcocks, Chidchamai Kewcharoenwong, Pattarachai Kiratisin, Peter W. Taylor, Brendan W. Wren, Ganjana Lertmemongkolchai, Richard A. Stabler, Jeremy Brown

**Affiliations:** ^1^Centre for Inflammation and Tissue Repair, University College London (UCL) Respiratory, London, United Kingdom; ^2^London School of Hygiene and Tropical Medicine, Infectious and Tropical Disease, Department of Infection Biology, London, United Kingdom; ^3^Cellular and Molecular Immunology Unit, Centre for Research and Development of Medical Diagnostic Laboratories (CMDL), Faculty of Associated Medical Sciences, Khon Kaen University, Khon Kaen, Thailand; ^4^Department of Microbiology, Faculty of Medicine Siriraj Hospital, Mahidol University, Bangkok-Noi, Bangkok, Thailand; ^5^School of Pharmacy, University College London, London, United Kingdom; ^6^Department of Medical Technology, Faculty of Associated Medical Sciences, Chiang Mai University, Chiang Mai, Thailand

**Keywords:** *A. baumannii*, antibodies, growth inhibition, neutrophils, heterologous protection

## Abstract

Antibody therapy may be an alternative treatment option for infections caused by the multi-drug resistant (MDR) bacterium *Acinetobacter baumannii.* As *A. baumannii* has multiple capsular serotypes, a universal antibody therapy would need to target conserved protein antigens rather than the capsular polysaccharides. We have immunized mice with single or multiple *A. baumannii* strains to induce antibody responses to protein antigens, and then assessed whether these responses provide cross-protection against a collection of genetically diverse clinical *A. baumannii* isolates. Immunized mice developed antibody responses to multiple protein antigens. Flow cytometry IgG binding assays and immunoblots demonstrated improved recognition of both homologous and heterologous clinical strains in sera from mice immunized with multiple strains compared to a single strain. The capsule partially inhibited bacterial recognition by IgG and the promotion of phagocytosis by human neutrophils. However, after immunization with multiple strains, serum antibodies to protein antigens promoted neutrophil phagocytosis of heterologous *A. baumannii* strains. In an infection model, mice immunized with multiple strains had lower bacterial counts in the spleen and liver following challenge with a heterologous strain. These data demonstrate that antibodies targeting protein antigens can improve immune recognition and protection against diverse *A. baumannii* strains, providing support for their use as an antibody therapy.

## Introduction

Antibiotic resistant *Acinetobacter baumannii* infections have spread rapidly across hospitals, particularly intensive care units, increasingly causing nosocomial infections that are associated with a mortality rate that can be greater than 50% (1–5). Clinical isolates of *A. baumannii* are often highly resistant to multiple antibiotics, including carbapenems, making them difficult to treat (5). Current treatment options use complex antibiotic combinations to overcome the antimicrobial resistance (AMR) profiles, but extremely multidrug resistant isolates with limited treatment options are now being identified in patients (6). This is compounded as currently, there are no licensed vaccines to counteract the growing threat of *A. baumannii* infections. As a consequence, *A. baumannii* is the top priority pathogen in recent WHO reports on AMR, and developing alternative methods of treating or preventing *A. baumannii* infections is a global imperative (1). *A. baumannii* seems to be a particularly prevalent pathogen in Asian countries: For example, in Thailand, infections with multi-drug resistant *A. baumannii* were the dominant cause of death due to AMR infections in 2010, estimated to cause over 25,000 deaths (7).

A potential adjunct treatment option for severe *A. baumannii* infections is monoclonal antibody therapy that specifically targets surface *A. baumannii* antigens, thereby improving immune recognition and clearance of the bacteria by the host (6, 8). Proof of principle studies have shown that whole *A. baumannii* cell preparations inactivated using either formalin (9, 10), antibiotic treatment (11), lipopolysaccharide removal (12), or by targeted gene deletion (13), induce antibody responses to *A. baumannii* that are partially protective against subsequent infections in mice with the homologous strain. Similarly, outer membrane complexes (OMCs) (14, 15), vesicles (OMVs) (16) and at least fifteen recombinant proteins (17–33) have been shown to elicit partially protective anti-*A. baumannii* antibody responses. However, the extensive genetic and antigenic heterogeneity in *A. baumannii* strains poses a significant challenge in the development of cross-protective antibody responses. Although anti-capsular antibody protects against *A. baumannii* infections in both mice (34) and rat (35) infection models, over 100 unique capsule loci (KL types) have been described for *A. baumannii* clinical strains (36). For example, a recent evaluation of 191 isolates collected from three tertiary hospitals in Thailand reported high capsule type diversity with a total of 24 KL types identified, and the three KL types with the highest prevalence KL10, KL6 and KL47 only accounting for 15.7%, 15.2% and 11.0% of the isolates evaluated respectively (37). This diversity inhibits using *A. baumannii* capsular antigen as the target for a monoclonal antibody therapy. Targeting conserved sub-capsular antigens offer an attractive alternative to targeting the capsular antigen. However, the relatively low abundance of protein antigens compared to capsular antigen and their sub-capsular localization suggests antibody to protein antigens are likely to be less protective than antibody to capsule (38). Furthermore, due to the extensive genetic diversity amongst *A. baumannii* strains, only approximately 2,000 genes are common to almost all strains, representing a minority of the over 20,000+ genes found in the *A. baumannii* pan-genome (39). Furthermore, only a small proportion of the core genes are predicted to be expressed on the surface of bacterial cells and therefore potentially accessible to therapeutic antibodies. Despite these problems, antibodies raised by vaccinating with either whole cells (9, 13, 14) or protein antigens (17, 20, 21, 29, 30, 40) have been shown to recognize and protect against infection with heterologous *A. baumannii* strains, indicating an anti-protein approach is feasible. However, the KL types and degree of genomic diversity between the strains used in these studies was unclear making the clinical potential of the demonstrated cross-protective immunity hard to assess.

Here we have investigated whether antibody responses developed in response to exposure to live *A. baumannii* are able to recognize heterologous strains, including those with a different KL type. To assess cross-reactivity between antisera and clinically relevant strains, we have tested mouse antisera raised against a single *A. baumannii* strain or three different strains against nine Thai clinical *A. baumannii* isolates. As antibody mediated immunity to bacterial pathogens can be mediated by improving bacterial agglutination, membrane attack complex (MAC)-dependent bacterial lysis or complement- or Fc*γ* receptor-dependent phagocytosis, we have used *in vitro* assays to evaluate these functions in different antisera. The protective efficacy of immunization with a selected combination of strains was tested in a mouse model of infection. Our hypothesis was that when compared to immunization with a single strain, sequential immunization with different clinical isolates would select for antibody responses to immunogenic, conserved *A. baumannii* protein antigens and thereby induce improved cross-protective antibody responses.

## Materials and Methods

### Bacterial Strains and Culture Conditions

The nine clinical *A. baumannii* isolates were obtained from patients admitted to Songklanagarind and Siriraj Hospitals, Thailand (37) (**Table 1**). AB5075 wild-type and unencapsulated AB5075^Δwza^ were obtained from the Manoil lab *A. baumannii* mutant library (https://www.gs.washington.edu/labs/manoil/baumanniii.htm). Bacteria were cultured at 37°C on LB plates or in LB broth to an optical density at 600nm of 0.8 (approximately 10^9^ CFU/ml) and stored at -80°C in 10% glycerol as single use aliquots.

**Table 1:** *A. baumannii* isolate information.

Strains	KL class	MLST type	Biological sample source	Hospital/Source
AB15**^*γ*^**	KL47	ST2	Sputum	Siriraj Hospital, Bangkok
AB98	KL47	ST215	Unknown	Siriraj Hospital, Bangkok
AB1615-09	KL47	ST164	Sputum	Songklanagarind Hospital, Songkhla
AB56	KL10	ST2	Unknown	Siriraj Hospital, Bangkok
AB3879**^*γ*^**	KL10	ST215	Sputum	Songklanagarind Hospital, Songkhla
AB1**^*γ*^**	KL52	ST2	Pus	Siriraj Hospital, Bangkok
NPRC-AB20	KL52	ST215	Unknown	Songklanagarind Hospital, Songkhla
AB55	KL6	ST2	Unknown	Siriraj Hospital, Bangkok
AB1492-09	KL2	ST2	Sputum	Songklanagarind Hospital, Songkhla
AB5075^WT^	KL25	ST1	n/a	Manoil lab *A. baumannii* mutant library
AB5075^Δwza^	n/a	ST1	n/a	Manoil lab *A. baumannii* mutant library

The nine Thai strains used in this study were isolated from patients across either the Siriraj or Songklanagarind hospitals in Thailand. The AB5075^WT^ and AB5075^Δwza^ strains lab isolates were obtained from the Manoil lab.

^*γ*^Strains were determined to be resistant to multiple classes of antibiotics that include a fluoroquinolone (n = 1), aminoglycosides (n = 2), a tetracycline (n = 1) and β-lactam antibiotics including carbapenems (n = 9). All three strains were sensitive to colistin (37).

### Bacteremia Mouse Model of *A. baumannii* Infection

All *in vivo* experiments using mice were performed according to United Kingdom national guidelines for animal use and care. Experiments performed at UCL were approved by the UCL Biological Services Ethical Committee and the UK Home Office (P64714548). Experiments used 5–6-week-old outbred female CD1 mice obtained from Charles River Laboratories. *A. baumannii* associated bacteremia mouse model was established by the intraperitoneal route by testing bacterial doses ranging from 10^6^ to 10^10^ CFU/mouse in the presence or absence of varying concentration of mucin. There was rapid clearance of bacteria at all doses when injected in the absence of mucin. A minimum lethal dose of 1.0 x 10^9^ CFU/mouse was determined for the AB1 strain. With this dose, significant target organ CFU could be recovered consistently at 24 hours post-inoculation. Mice rapidly developed fatal infection at later time points. To evaluate the protective efficacy of the AB3879 and multi strain antisera, the AB1 stock was washed and resuspended in appropriate volume of 5% Mucin in PBS to inject a dose of 6.0 x 10^9^ CFU/per mouse. Groups of mice injected with 5% Mucin/PBS served as a control. At 24 hours post infection, mice were sacrificed, and the lungs, liver and spleen harvested, homogenized and the bacterial counts obtained by plating serial dilutions on LB agar plates.

### Anti*-A. baumannii* Mouse Sera

The majority of experiments were performed using pooled serum obtained from mice immunized with live sub-lethal doses (10^6^ CFU/mouse/vaccination) of *A. baumannii via* the intraperitoneal route. Homologous sera namely anti-AB3879, anti-AB5075^WT^ and anti-AB5075^Δwza^ were generated following three immunizations, 14 days apart with the same *A. baumannii* isolate. Multi strain antisera was generated by sequential immunization with AB3879, AB1492-09 and AB1615-09 isolates respectively. Sera from non-immunized mice were used as controls.

### Whole Cell ELISA

Antibodies specific to antigens in different *A. baumannii* strains were measured by whole cell ELISA. Briefly, *A. baumannii* were grown to late log-phase (OD_600nm_ = 0.8, approximately 10^9^ CFU/ml), washed, and resuspended in PBS. 96-well plates (Brand plates, IMMUNOGrade) were coated with 50 µl of this bacterial suspension, overnight at 4°C, inactivated using 50 µl of 4% paraformaldehyde for 10 minutes, then blocked with 5% non-fat milk/0.05% Tween 20/PBS (blocking buffer) for 3 hours at room temperature. Sera were diluted to a 1:100 dilution in blocking buffer before addition, and binding to bacterial antigens detected with goat anti-mouse IgG conjugated to Horse-radish peroxidase (Invitrogen) and detected using the TMB substrate.

### Immunoblot Detection of Bacterial Lysate Recognition by IgG

*A. baumannii* bacterial lysate were obtained by resuspending bacteria in 1X lithium dodecyl sulfate (LDS) sample buffer (NuPAGE LDS Sample buffer (4X), Invitrogen) with 50mM dithiothreitol [NuPAGE sample reducing agent (10X)], boiled at 95°C for 10 min, separated on a gradient 4-12% SDS-polyacrylamide gel (NuPAGE Bis-Tris gels), and transferred to nitrocellulose membranes using the iBlot 2 Dry Blotting System (ThermoFisher Scientific). Nitrocellulose membranes were blocked with 10% non-fat milk/0.05% Tween 20/PBS (blocking buffer) for 2 hours then incubated with mouse sera at a 1:1000 dilution overnight at 4°C, washed and then probed using an anti-mouse IgG conjugated with horseradish peroxidase. Protein bands were visualized using the chemiluminescent ECL reagent (Amersham) on the ImageQuant™ LAS 4000.

### Flow-Cytometry Detection of Bacterial Surface Recognition by IgG

Total IgG binding to *A. baumannii* was assessed using flow cytometry. 10^6^ CFU of *A. baumannii* was incubated with mouse antisera in triplicate for 1 hour at 37°C, followed by goat anti-mouse IgG-allophycocyanin (APC) (Invitrogen) for 1 hour at room temperature. Samples were analyzed using a BD FACSVerse and data processed using FlowJo software for Windows (version 10). Markers for identifying IgG positive bacteria was set using bacteria incubated with PBS and then incubated with the secondary antibody. The proportion of IgG positive bacteria was gated on the single-cell population of unstained and bacteria labelled with secondary antibody only. Two independent experiments were conducted using stocks cultured on separate days and stored as single-use aliquots. A positive correlation between the two independent experiments was observed (Spearman correlation: R=0.7157, p-value=0.0359).

### Genomic Analysis of the Capsule Locus

Capsule KL loci were previously determined using Kaptive and an *A. baumannii* KL database (37, 41).

### Growth Inhibition Assays

10^2^ CFU of *A. baumannii* was incubated with 1:100 dilution of mouse antisera in triplicate and bacterial growth (OD_600nm_) at 37°C in 5% C02 was monitored every hour over a 24-hour period using the Spark multimode microplate reader (TECAN).

### Neutrophil Opsonophagocytosis Assays

Phagocytosis was investigated using an established flow cytometry assay (42), neutrophils extracted from fresh human blood from healthy adult donors and fluorescent *A. baumannii* labeled with 6-carboxyfluorescein succinimidyl ester (FAMSE, Molecular Probes). Bacteria was opsonized with mouse antisera and 5% heat-inactivated human sera for 30 minutes at 37°C followed by the addition of 10^5^ neutrophils to a final multiplicity of infection (MOI) of approximately 1:100. A minimum of 5000 cells were analyzed by flowcytometry using a BD FACSVerse and data processed using FlowJo software for Windows (version 10). To identify the percentage of neutrophils associated with bacteria, neutrophils that had not been incubated with bacteria were used to identify the negative control. The proportion of bacteria positive neutrophils was gated on the single-cell population of neutrophils not incubated with bacteria. To combine the percentage of bacteria associated neutrophils and the intensity of association, a fluorescence index was determined by multiplying the percentage of positive neutrophils by the geometric median fluorescence of the positive population. All sera were tested in parallel on the same day, at the same concentration with the same batch of bacteria and neutrophils for each independent experiment.

### Bacterial Cell Size Measurement

*A. baumannii* strains were cultured in LB broth to an OD_600nm_ of 0.4, pelleted and resuspended in 300 µL sterile PBS. 10 µL bacteria solution was mixed with 2 µL 2000 kDa FITC-Dextran (Sigma) at 10 mg/ml before spotting 10 µL onto a glass microscope slide and sealed with a coverslip. Slides were imaged using a Zeiss LSM 880 confocal microscope with Plan-Apochromat 63x/1.4 Oil DIC M27 using standard confocal mode driven by ZEN Black 2.3 software (Carl Zeiss). A minimum of 1,000 individual bacteria were imaged using a 490 nm laser with collection of light at 520 nm. To measure bacterial cell length, ImageJ 1.53a software was used to first convert images to 8-bit with auto-threshold, before identifying singlet bacterial particles within a defined circularity and size range.

### Statistical Analysis

Statistical analyses were conducted using GraphPad Prism version 8 (GraphPad, USA). Data are presented as means, and the error bars represent standard deviations. Parametric data were analyzed using Student’s T test and nonparametric data using the Mann-Whitney U test. Doubling times from growth curves were calculated using the log of exponential growth equation in GraphPad version 8 (https://www.graphpad.com/guides/prism/latest/curve-fitting/reg_log-of-exponential-growth.html). The optical density values obtained from bacterial growth at the exponential phase (6-16 hours) were log transformed and the non-linear regression of exponential growth with a log population equation used to calculate doubling time in the presence of respective anti-*A. baumannii* sera. The doubling time ratio was calculated by dividing doubling time in respective sera over doubling time in sera from non-immunized mice (DT sera/DT control).

## Results

### Immunization With *A. baumannii* Induces Antibody Responses That Recognize Multiple Conserved Proteins

Nine *A. baumannii* strains were isolated in 2016 from clinical specimens obtained from two tertiary hospitals in Thailand, Siriraj Hospital, Bangkok and Songklanagarind Hospital, Songklanagarind Hospital, Hat Yai, Songkhla Province. These strains subsequently underwent whole-genome sequencing and sequence typing (37). The nine isolates tested had a range of multilocus sequence typing (MLST) sequence types (ST) and KL types including five ST2 (representing global clone 2), three ST215 and one ST164 strains and three KL47, two KL10, two KL52, one KL6 and one KL2 capsular loci strains (**Table 1**). None of the isolates share both the capsule loci and MLST (Institute Pasteur Scheme) type. Two additional strains, the AB5075 (ST2, KL25) wild-type and the unencapsulated AB5075^Δwza^ (strain name: tnab1_kr121203p06q153, genotype: *wza*-153::T26) strain were obtained from the Manoil lab *A. baumannii* mutant library (https://www.gs.washington.edu/labs/manoil/baumanniii.htm).

**Figure 1:**
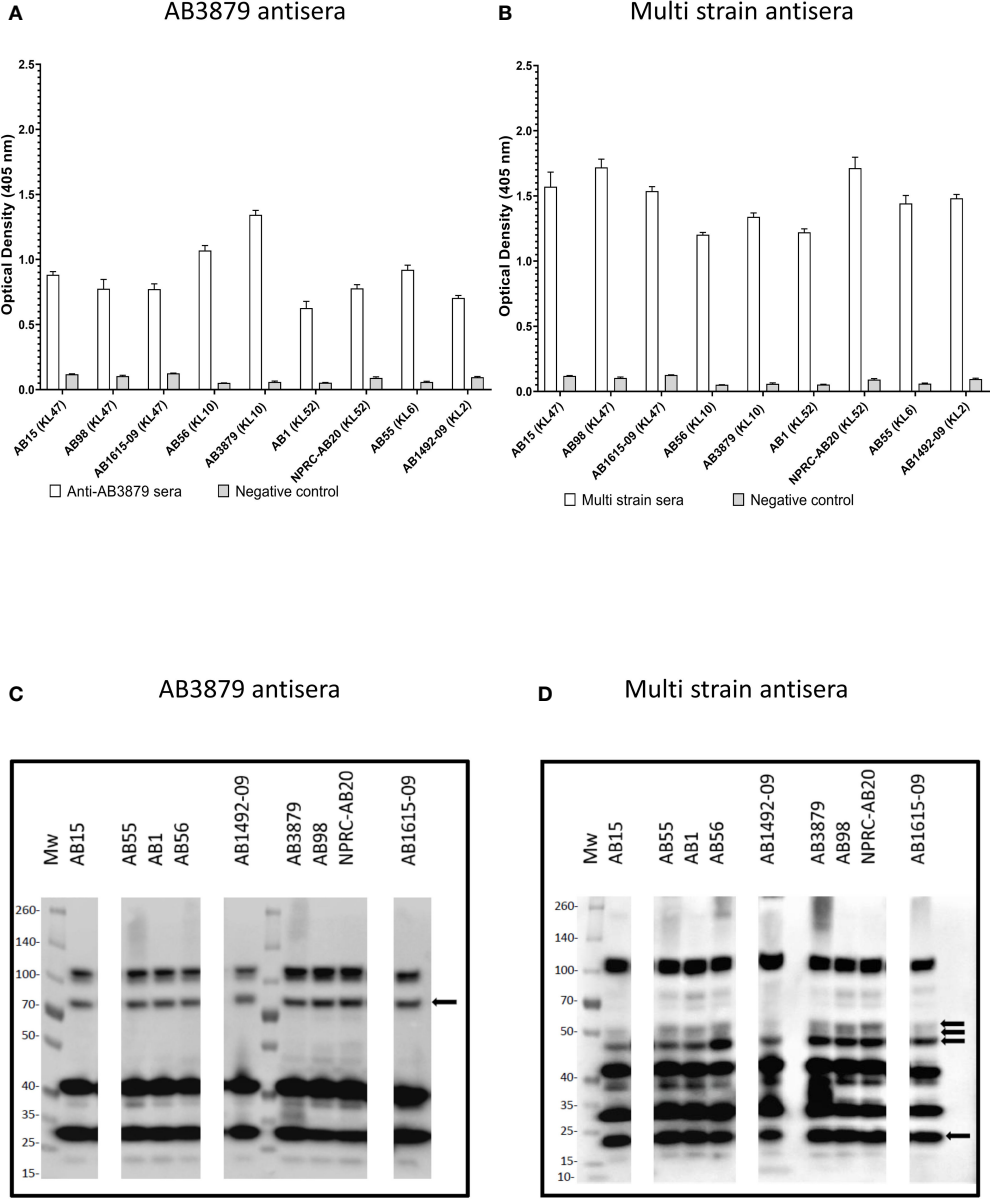
Whole cell ELISA and immunoblot detection of the reactivity of mouse antisera with *A. baumannii* isolates. Whole cell ELISAs of IgG binding to nine *A. baumannii* isolates using either AB3879 **(A)** or multi strain antisera **(B)**. Whole cell lysates from the nine diverse *A. baumannii* isolates were resolved on a reduced 4-12% gradient gel, probed with mouse antisera and anti-mouse IgG-HRP. Each lane represents a clinical *A. baumannii* isolate indicated above. Immunoreactive proteins detected when blot was probed with either AB3879 **(C)** or multi strain antisera **(D)**. Black arrows represent bands unique to immunoblots probed with either AB3879 or multi strain sera. Bars represent mean values for each group and the error bars indicate standard deviations (SDs) (n = 3). Representative data from two independent experiments is shown.

Groups of mice were immunized with either AB3879 (KL10 type) or sequentially with the AB3879, AB1492-09 (KL2) then AB1615-09 (KL47) strains (multi strain group) with both groups receiving the same total bacterial CFU. Sera was obtained 2 weeks after the third injection, pooled and used for whole cell ELISAs and to probe immunoblots of all nine *A. baumannii* strains. Whole cell ELISAs showed both antisera recognized all nine strains to varying degrees, with higher antibody levels to all strains observed with the multi strain antisera (**Figures 1A, B**). The immunoblots showed both antisera recognized multiple bands of similar size for all the probed *A. baumannii* strains (**Figures 1C, D**). More immunoreactive bands were visible in immunoblots probed with multi strain antisera (**Figure 1D**, black arrows), although one band above 70 KDa was weaker compared to the results for lysates probed with AB3879 antisera (**Figure 1C**, black arrow). These data demonstrate that mouse antisera generated by inoculation of live *A. baumannii* can recognize different *A. baumannii* strains expressing different KL types and from different STs, and that at least some of the antibody responses target multiple protein antigens that are likely to be conserved between *A. baumannii* strains.

### The Effects of the Capsule on Antibody Binding to *A. baumannii* in Sera From Immunized Mice

**Figure 2:**
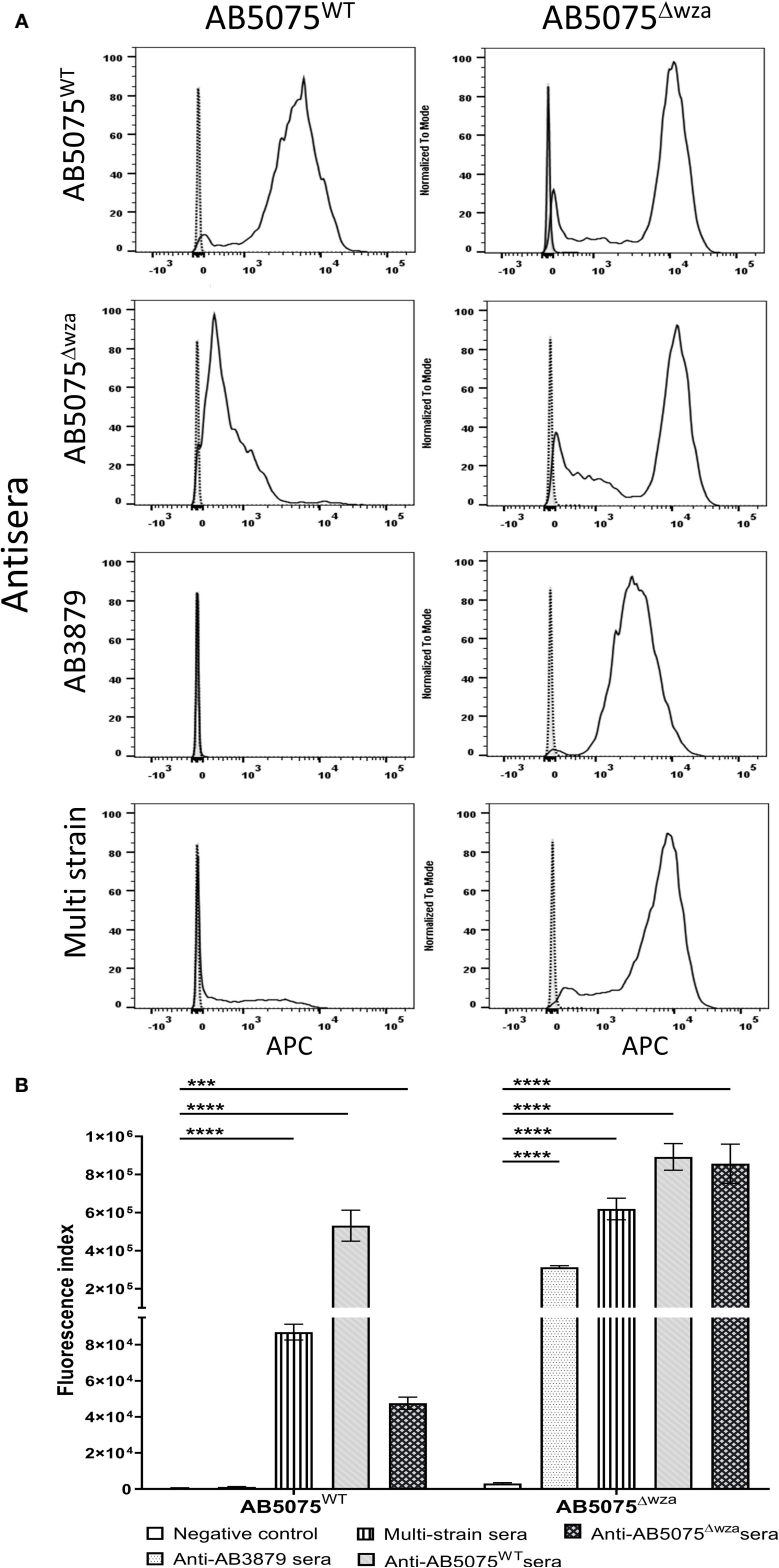
Flow cytometry detection of IgG binding to wild type and un-encapsulated AB5075 isolates by mouse antisera. Representative histograms showing the recognition of 1x10^6^ CFU of AB5075 wild-type strain (left panel) and un-encapsulated AB5075^Δwza^ strain (right panel) to either **(A)** AB5075^WT^, AB5075^Δwza^, AB3879 or multi strain antisera. **(B)** Bar graph presenting the proportion of AB5075^WT^ and AB5075^Δwza^ bacteria bound by IgG in AB3879, multi strain, AB5075^WT^ or AB5075^Δwza^ antisera. The Y axis shows the fluorescence index calculated by multiplying the percentage of IgG positive bacteria to the median fluorescent intensity of the positive population that was detected in each isolate. Bars represent mean values for each condition/strain and the error bars indicate standard deviations (SDs) (n = 3). T-test was used for statistical analysis ***p-value < 0.001, ****p-value =< 0.0001. Representative data from two independent experiments is shown.

Previous authors have demonstrated that the *A. baumannii* polysaccharide capsule inhibits access of antibody to underlying protein antigens (18). In addition, antisera raised in mice to the wild-type *A. baumannii* strains is also likely to contain a mixture of IgG to capsular and protein antigens. To assess whether the *A. baumannii* capsule was a target antigen to IgG in the antisera and/or inhibited anti-protein antigen recognition, we used a flow cytometry IgG binding assay to measure IgG deposition on live *A. baumannii* incubated in antisera. Initially, we measured IgG deposition on the AB5075 wild-type (KL25, ST1) and unencapsulated AB5075^Δwza^ strains using antisera raised by immunizing mice with either of these two strains. This confirmed that mouse antisera raised against encapsulated *A. baumannii* contained IgG that recognized the capsule, with increased IgG deposition on the AB5075 wild-type strain when incubated in antisera raised against the encapsulated AB5075 wild-type compared to the unencapsulated AB5075^Δwza^ strain (**Figure 2A**, left panel and B). The results also indicated there was significant IgG in both AB5075^WT^ and AB5075^Δwza^ antisera against protein antigens, with marked IgG deposition occurring on the AB5075^Δwza^ unencapsulated strains (**Figure 2A**, right panel and B). When incubated in antisera raised against the AB5075^Δwza^ unencapsulated strain, there was reduced IgG deposition on the AB5075 strain compared to the AB5075^Δwza^ strain demonstrating the capsule impaired recognition of *A. baumannii* by IgG to protein antigens (**Figure 2A**, 2^nd^ row). When incubated in AB3879 and multi strain antisera there were high levels of IgG binding to the unencapsulated AB5075^Δwza^ strains, confirming these sera contained a significant anti-protein antigen IgG response (**Figures 2A, B**). However, there was markedly reduced IgG binding to the encapsulated AB5075 wild-type strain in AB3879 and multi strain antisera, confirming the capsule impaired recognition of protein antigens. There was a small degree of residual IgG binding to the encapsulated AB5075 wild-type strain in the multi strain antisera, demonstrating that some recognition mediated by antiprotein IgG occurred in this serum even against an encapsulated strain.

### IgG Binding to Diverse *A. baumannii* Strains Incubated in Antisera From Immunized Mice

**Figure 3:**
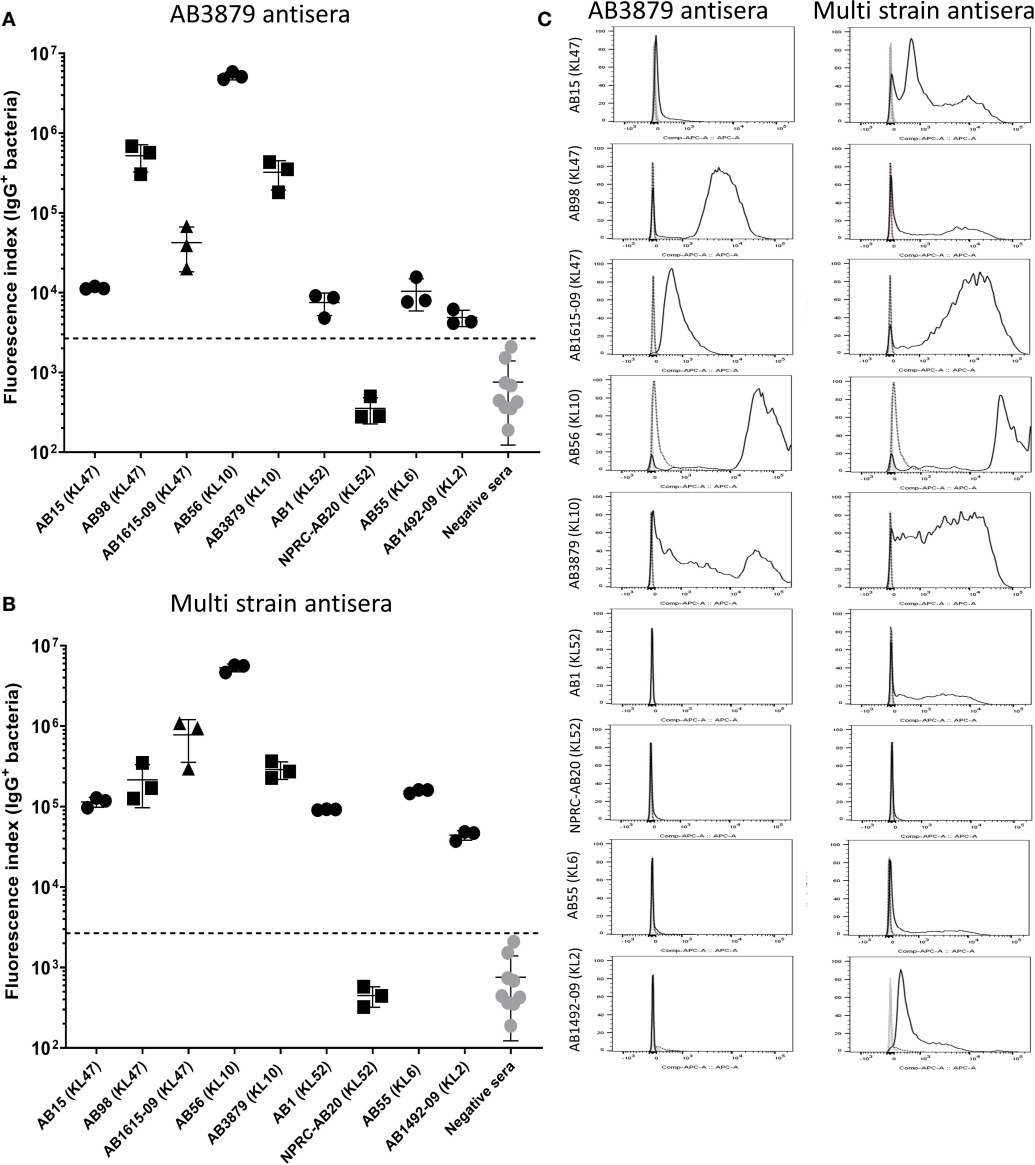
IgG recognition of a panel of nine *A. baumannii* isolates by mouse antisera generated against immunization with one strain or sequentially with three strains. **(A)** Mouse AB3879 antisera and **(B)** multi strain (AB3879+AB1492-09+AB1615-09) antisera IgG binding to the surface of *A. baumannii* isolates as measured by flow cytometry. The nine isolates are grouped in order of their respective capsule serotypes and are indicated in brackets on the X-axis. Round, square and triangle isolates belong to the ST2, ST215 and ST164 MLST type respectively. The Y axis shows the proportion of IgG positive bacteria multiplied by the median fluorescent intensity (MFI) of IgG positive population, in each of the nine *A. baumannii* isolates. **(C)** Representative histograms showing IgG binding of 1x10^6^ CFU of the nine *A. baumannii* isolates to either AB3897 antisera (left panel) or multi strain antisera (right panel). Grey histogram represents bacteria labelled with 2^nd^ antibody only, black-dotted line represents bacteria incubated with sera from non-immunized mice and black solid line represents bacteria incubated with the respective sera (AB3879 antisera left panel and multi strain antisera right panel). The nine isolates and their respective KL class denoting the capsule serotypes are indicated alongside respective histograms. Dot plots represent triplicate values with lines indicating the mean values and the error bars indicate standard deviations (SDs) (n = 3). Representative data from two independent experiments is shown.

**Figure 4:**
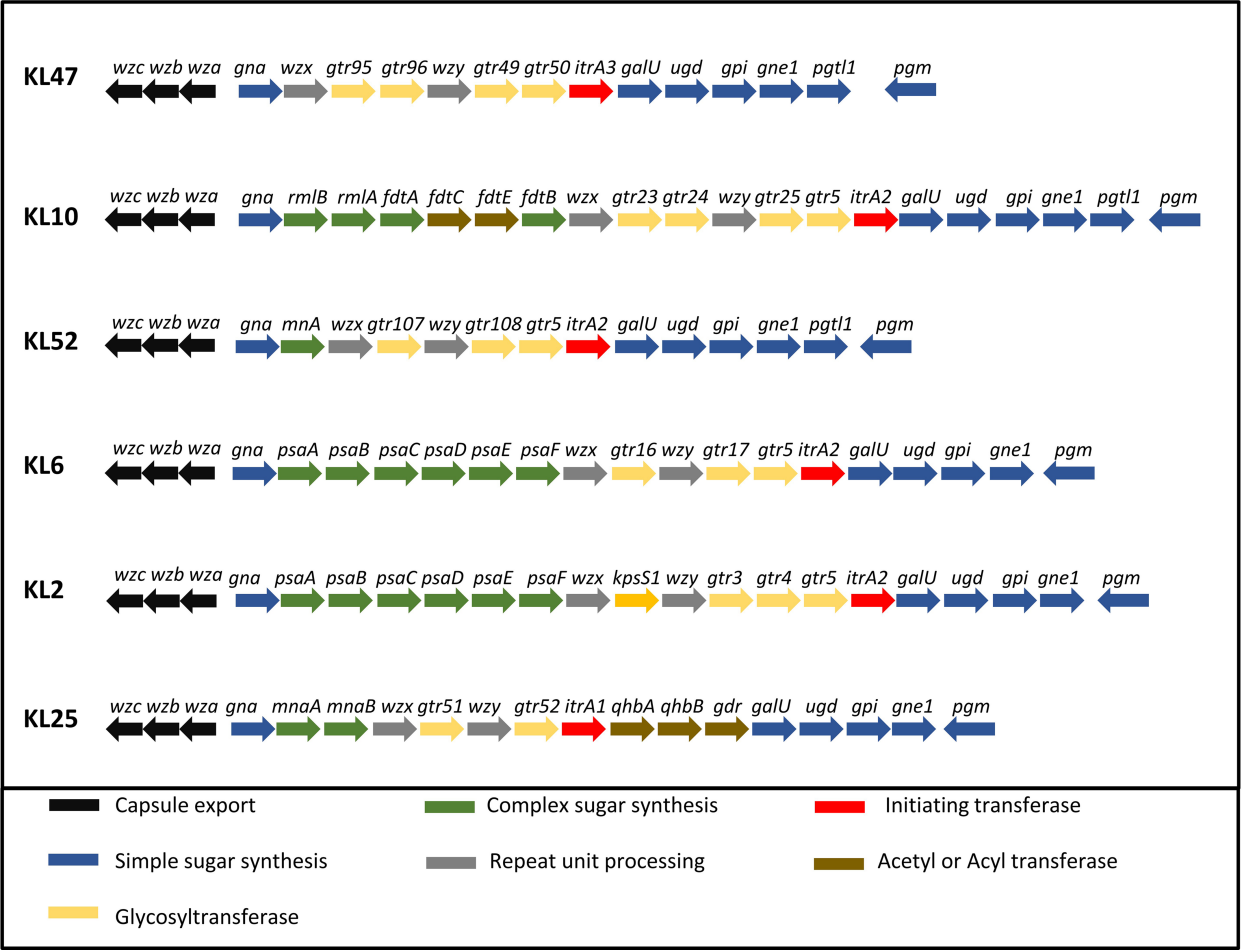
Organization of the five KL loci represented in the *A. baumannii* strains. A comparison of the CPS biosynthesis genes clusters and their organization in the five KL loci represented in the *A. baumannii* strains. CPS export genes are in black, genes in dark blue are involved in the synthesis of common sugar substrates, genes in green are involved in the synthesis of complex sugars, genes in yellow encode glycosyltransferases, genes in red encode initiating transferases and genes in brown encode Acyl or acetyl transferases.

To further characterize whether capsular and/or protein antigens were targets for IgG in the AB3879 and multi strain antisera, the IgG deposition assay was repeated with all nine Thai clinical isolates. For the AB3879 antisera, the highest levels of IgG binding occurred to the homologous AB3879 strain, and to strain AB56 that shares the same capsule locus (KL10) (**Figures 3A, C**). In addition, after incubation in AB3879 antisera, there was significant IgG binding to two of the KL47 capsule strains (AB98 and AB1615-09), one of which shared the same ST type to AB3879 (ST215). There was minimal IgG binding to the remaining strains (**Figures 3A, C**). These data show that although the sera to AB3879 contains antibodies to multiple conserved protein antigens, IgG deposition in this antiserum correlated with strain KL type except for the KL47 strains. Genomic analysis of the capsule loci of the Thai clinical isolates showed significant differences between the KL47 and KL10 capsule loci, suggesting that preservation of capsule structure between these KL types is unlikely to explain the cross-reactivity observed (**Figure 4**).

In contrast to the data for AB3879 antisera, incubation in multi strain antisera (raised against KL10, KL2 and KL47 strains) resulted in IgG binding to eight of the nine strains investigated (**Figures 3B, C**). The highest binding was to AB3879 (KL10) and AB1615-09 (KL47) strains included in the vaccination schedule, as well as the KL10 strain, AB56 (similar to the results for AB3878 antisera), to two other KL47 strains and the AB1492-09 (KL2) strain (included in the vaccination schedule). In addition, there were also lower levels of IgG binding detectable to the heterologous strains AB1 (KL52) and AB55 (KL6) (**Figures 3B, C**) indicating that anti-protein responses in multi strain antisera allows immune recognition of a wider range of strains than in the AB3879 antisera.

### Effects of Antisera on Growth of Encapsulated AB5075^WT^ and Unencapsulated AB5075^Δwza^ *A. baumannii* Strains

**Figure 5:**
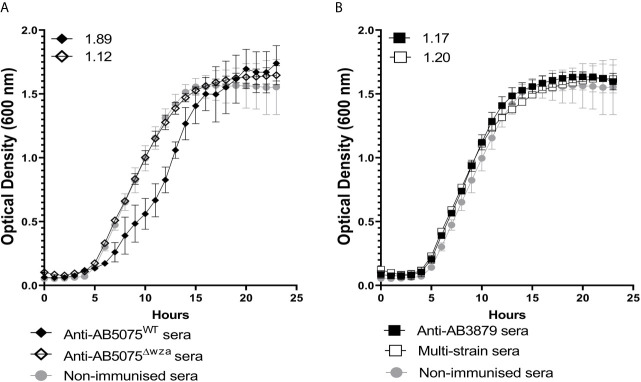
Growth inhibitory activity of wild type AB5075 isolates by mouse antisera. 10^2^ CFU of encapsulated AB5075^WT^strain was incubated with either, AB5075^WT^ antisera (black line, solid diamond), AB5075^Δwza^ antisera (black line, open diamond) and sera from non-immunized mice (grey line, solid circle) **(A)** or AB3879 antisera (black line, solid square), multi strain antisera (black line, open square), and sera from non-immunized mice (grey line, solid circle) **(B)**. Numbers on the top left corner represent the ratios of doubling time for respective antisera. Ratios of doubling time in test sera/control sera were calculated with a ratio of > 1.2 indicating delayed growth in the antisera. Line graphs indicate the mean value, and the error bars indicate standard deviations (SDs) (n=6).

Agglutination by specific antisera can inhibit bacterial growth in broth culture (43). To evaluate whether anti-capsular and/or anti-protein antibodies could inhibit *A. baumannii* growth, we used antisera raised against the encapsulated AB5075 wild-type and the unencapsulated AB5075^Δwza^ strain to measure growth inhibition in both AB5075^WT^ and AB5075^Δwza^ strains. Growth of the encapsulated AB5075 wild-type strain was inhibited by antisera raised against the encapsulated AB5075 strain but not by antisera raised against the unencapsulated AB5075^Δwza^ or AB3879 or the multi strain antisera (**Figure 5**). Reduced growth of the unencapsulated AB5075^Δwza^ strain was only observed when approaching the stationary phase after incubation with antisera raised against AB5075^WT^, AB5075^Δwza^ and the multi strain group (**Supplementary Figure 1**), suggesting late phase growth could be inhibited to a small degree by anti-protein antibodies. These data indicate growth inhibition was largely dependent on capsule specific antibodies.

### Growth Inhibitory Activity of AB3879 and Multi Strain Antisera Against Thai *A. baumannii* Strains

**Figure 6:**
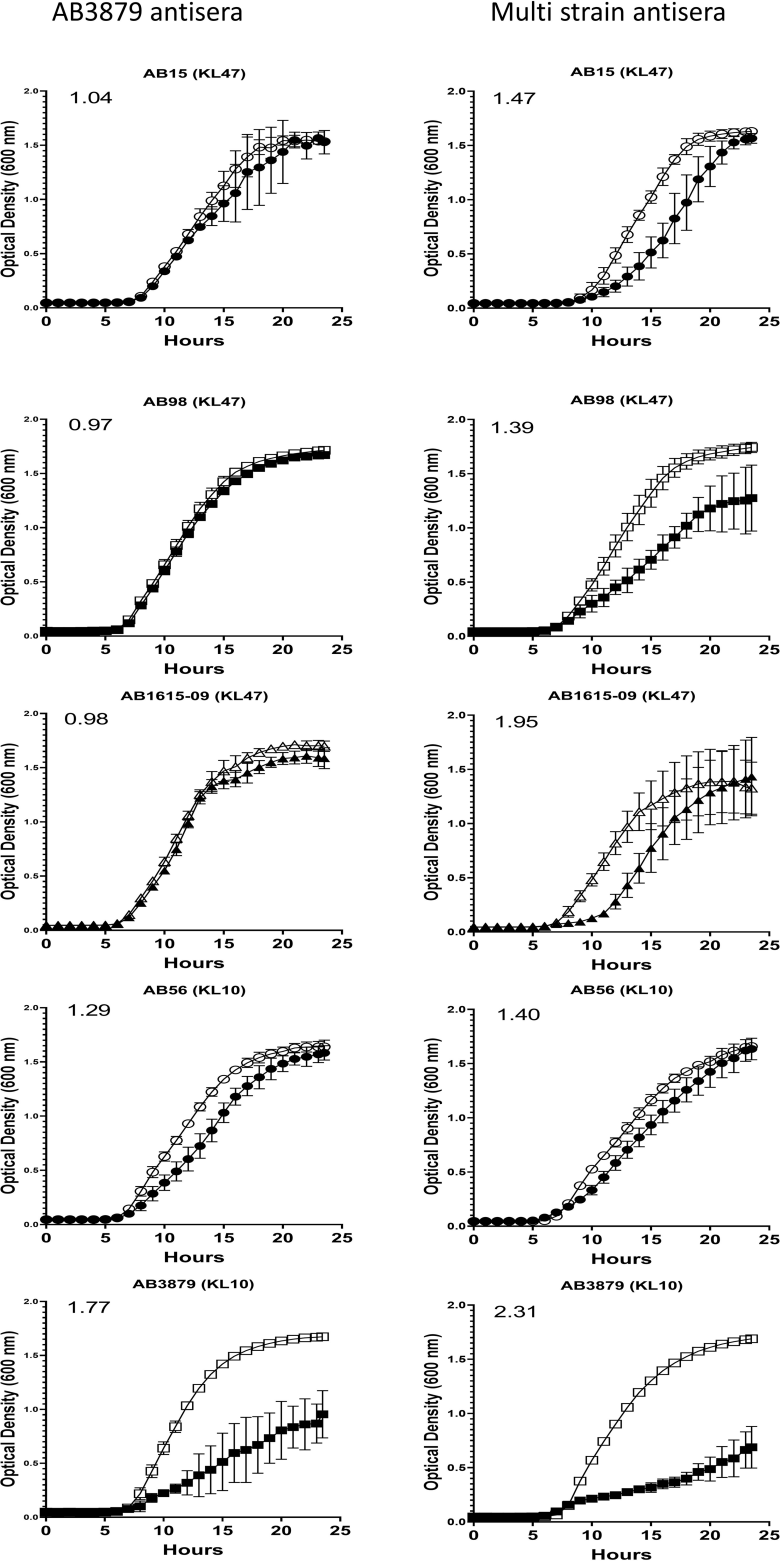
Growth inhibitory activity on a panel of five *A. baumannii* isolates by mouse antisera. 10^2^ CFU of a panel of *A. baumannii* isolates were incubated in triplicate with either AB3879 antisera (left panel) or multi strain antisera (right panel) and the OD_600nm_ measured every 30 minutes for 24 hours. Sera from non-immunized mice was included as a control (open symbols). Solid symbols represent respective anti-*A. baumannii* antisera. The five isolates are grouped by their capsule serotype and indicated at the top of each graph in brackets. Round, square and triangle symbols represent ST2, ST215, and ST164 MSLT types respectively. Numbers on the top left-hand corner represent the ratios of doubling time for respective antisera. Ratios of doubling time in test sera/control sera were calculated with a ratio of > 1.2 indicating delayed growth in the antisera. Line graphs indicate the mean value, and the error bars indicate standard deviations (SDs) (n = 6).

To further characterize the inhibitory activity in the AB3879 and multi strain antisera, the growth inhibition assay was repeated with all nine Thai clinical isolates. When compared to sera from non-immunized mice, AB3879 antisera inhibited the growth of both KL10 strains AB3879 and (to a lesser extent) AB56, with an increased doubling time and significantly reduced the total CFU reached in culture for both strains (**Figure 6** and **Table 2**). Multi strain antisera (raised against KL10, KL2 and KL47 type strains) inhibited growth and increased the doubling times for all five KL10 or KL47 strains, and significantly reduced the total CFU reached for the AB98 (KL47), AB1615-09 (KL47) and AB3879 (KL10) strains (**Figure 6** and **Table 2**). Overall, all the strains showing significant growth inhibition in *A. baumannii* antisera were the homologous KL types to strains used to generate the antisera and showed high levels of IgG binding in the flow cytometry assay (**Figure 3**), suggesting this effect was largely dependent on anti-capsular antibodies. However, growth was not inhibited in antisera for every strain showing high levels of IgG binding (e.g., AB98 and AB1615-09 in AB3879 antisera) or for all KL types used to generate the antisera (e.g., the KL2 strain AB1492-09 in multi strain antisera) (**Supplementary Figure 2**).

**Table 2:** Total CFU counts to measure the growth inhibitory effects of AB3879 antisera and multi strain antisera.

Strains	KL class	ST type	Viable bacterial CFU^¥^		
			antisera	control	P-value
**AB3879 antisera**					
AB56	KL10	ST2	2.67 ± 1.66 x 10^8^	1.38 ± 1.12 x 10^9^	**0.017**
AB3879	KL10	ST215	3.33 ± 1.83 x 10^7^	7.20 ± 6.97 x 10^8^	**0.002**
**Multi strain antisera**					
AB15	KL47	ST2	5.50 ± 7.33 x 10^8^	5.83 ± 4.90 x 10^8^	0.485
AB98	KL47	ST215	3.25 ± 0.69 x 10^8^	5.25 ± 2.40 x 10^8^	**0.039**
AB1615-09	KL47	ST164	7.75 ± 2.52 x 10^7^	3.87 ± 1.95 x 10^8^	**0.015**
AB56	KL10	ST2	2.75 ± 1.86 x 10^8^	4.08 ± 0.86 x 10^8^	0.130
AB3879	KL10	ST215	3.83 ± 1.40 x 10^7^	6.95 ± 5.06 x 10^8^	**0.002**

10^2^ CFU of a panel of A. baumannii isolates was incubated in triplicate with either pooled mouse AB3879 or multi strain antisera at a 1:100 dilution. Growth in sera from non-immunized mice was included as the control. Viable bacterial counts of isolates were determined by CFU counts after 14 hours of incubation. Bold p-values indicate statistically significant differences in bacterial counts.

^¥^Pooled data from 2 independent experiments. Each sample tested in triplicate in each experiment (N = 6).

### The Effects of the Capsule on Promotion of *A. baumannii* Neutrophil Phagocytosis by Antisera

**Figure 7:**
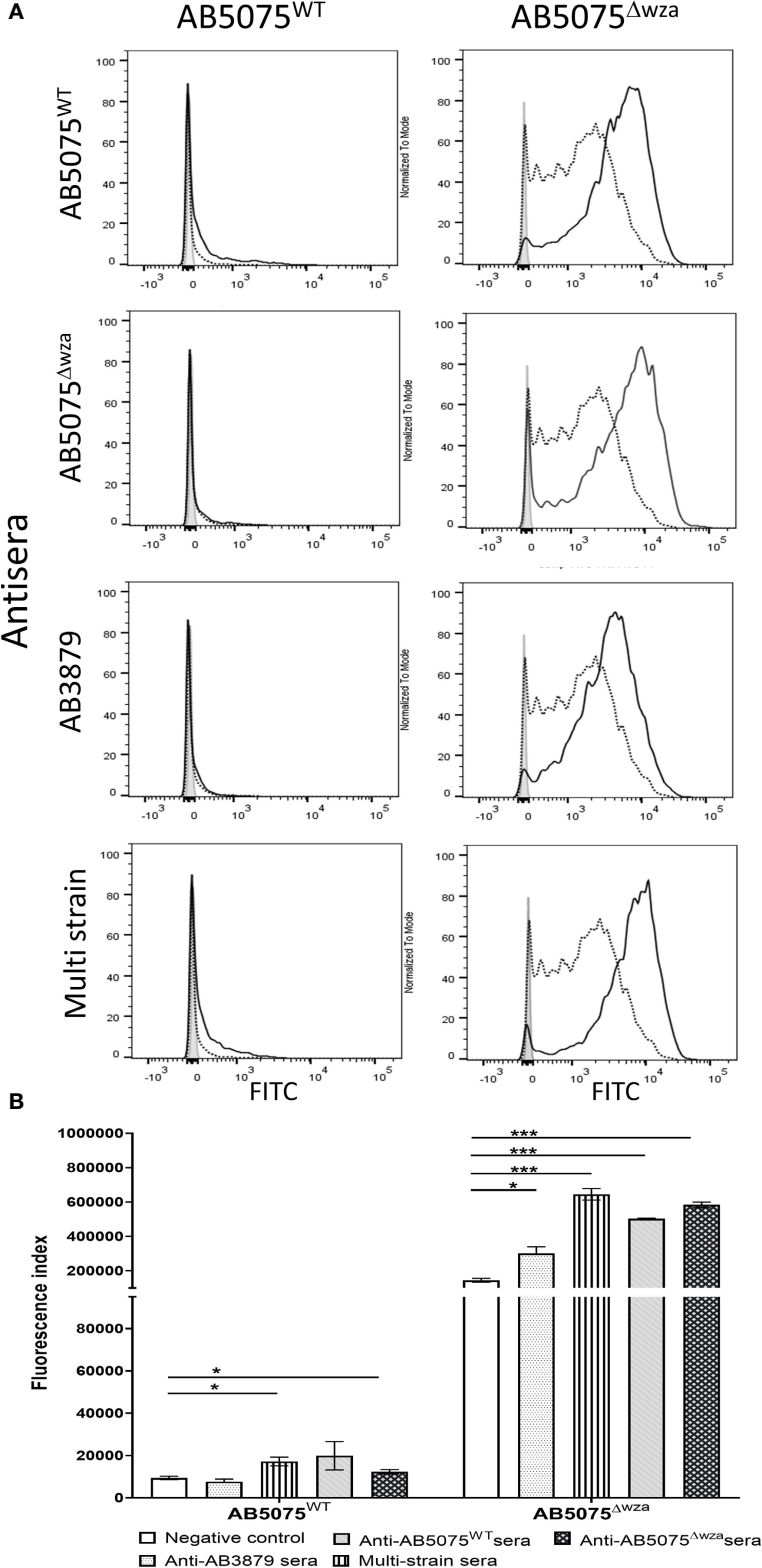
Neutrophil opsonophagocytosis of wild type and un-encapsulated AB5075 isolates by mouse antisera. **(A)** FAMSE labelled AB5075^WT^ (left panel) and AB5075^Δwza^ (right panel) were opsonized with either AB5075^WT^, AB5075^Δwza^, AB3879 or multi strain antisera and incubated with healthy human neutrophils at Bacteria: Neutrophil MOI of 100:1. Sera from non-immunized mice was included as a control. Each graph shows a grey histogram that represents neutrophils incubated with no sera, black-dotted line represents neutrophils with bacteria opsonized with sera from non-immunized mice and black solid line represents neutrophils with bacteria opsonized with the respective sera indicated on the left. **(B)** Bar graph showing fluorescence index calculated by multiplying the percentage of IgG positive bacteria to the median fluorescent intensity of the positive population that was detected in each isolate, on the Y axis. The X-axis shows either AB5075^WT^ (left) or AB5075^Δwza^ (right) opsonized with either AB3879, multi strain, AB5075^WT^ or AB5075^Δwza^ antisera as indicated in the legend. Bars represent mean values for each condition/strain tested in triplicate and the error bars indicate standard deviations (SDs) (n=3). T-test was used for statistical analysis *p-value < 0.05, ***p-value < 0.001 Representative data from two independent experiments is shown.

To assess whether the *A. baumannii* capsule inhibited neutrophil phagocytosis following opsonization with antisera, we measured phagocytosis using a flow cytometry assay of the encapsulated AB5075 wild-type and the unencapsulated AB5075^Δwza^ strains using antisera raised by immunizing mice with either of these two strains. As expected, the encapsulated AB5075 strain was considerably less susceptible to neutrophil phagocytosis compared to the unencapsulated AB5075^Δwza^ strain following opsonization with all antibodies, including sera from non-immunized mice (**Figure 7**). Although IgG deposition on the AB5075 wild-type strain was observed with antisera raised against AB5075^WT^, AB5075^Δwza^ and the multi strain group (**Figure 2**), only incubation in AB5075^WT^ and to a lesser extent multi strain antiserum, increased neutrophil phagocytosis of this strain (**Figures 7A, B**). In contrast, the unencapsulated AB5075^Δwza^ strain showed significant increases in susceptibility to neutrophil phagocytosis following opsonization with all antisera tested. Cumulatively, these data confirm the *A. baumannii* capsule inhibits promotion of neutrophil phagocytosis by antisera, but despite this, anti-protein antibodies in the multi strain antisera can promote a modest improvement of the phagocytosis of a heterologous encapsulated strain.

### Effects of Incubation in Antisera on Phagocytosis of Thai *A. baumannii* Strains

**Figure 8:**
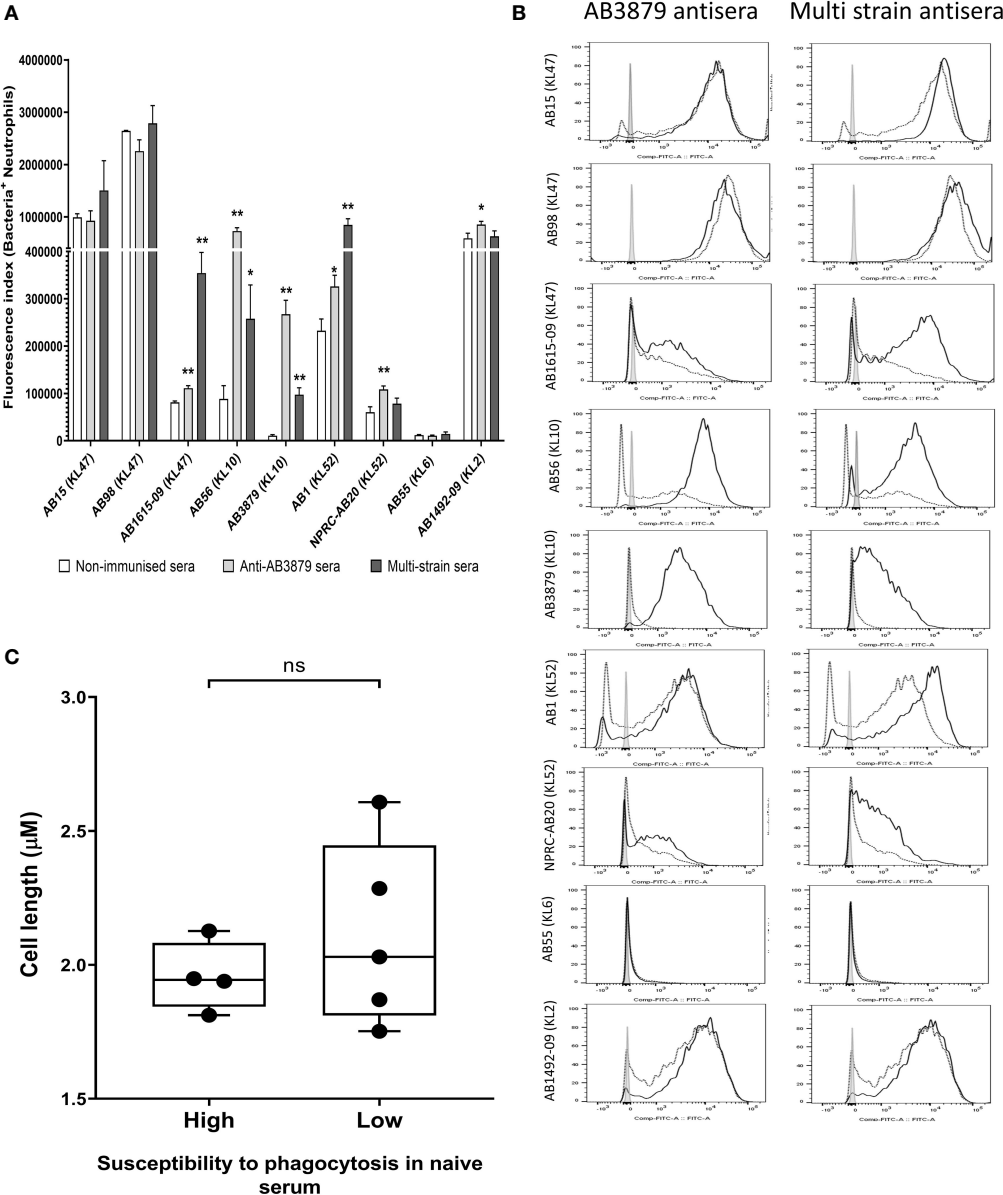
Opsonophagocytosis by human neutrophils of a panel of nine *A. baumannii* isolates by mouse antisera generated following immunization with one strain or sequentially with three strains. Nine FAMSE labelled *A. baumannii* isolates were opsonized with either AB3879 antisera, multi strain antisera or sera from non-immunized mice and incubated with healthy human neutrophils at Bacteria: Neutrophil MOI of 100:1. **(A)** Shows the fluorescence index calculated by multiplying the proportion (%) of FAMSE^+ve^ neutrophils and the median fluorescence intensity of the positive population [mean (± standard deviation)] on the Y axis and the nine isolates with their capsule serotypes indicated in brackets are on the X-axis. Phagocytosis indices in bacteria incubated with non-immune sera, anti-AB3879 sera and multi strain sera for each isolate is represented in the white, grey and dark grey bars respectively. Bars represent mean values for each condition/strain tested in triplicate and the error bars indicate standard deviations (SDs) (n = 3). T-test was used for statistical analysis *p-value < 0.05, **p-value < 0.001, ***p-value < 0.0001, ns: p-value= not significant. Representative data from two independent experiments is shown. **(B)** Representative histograms showing phagocytosis of FAMSE labelled *A. baumannii* isolates incubated with either AB3897 antisera (left panel) and multi strain antisera (right panel). Grey histograms represent no sera (neutrophils only), black-dotted line represents addition of neutrophils to bacteria incubated with sera from non-immunized mice and black solid line represents addition of neutrophils to bacteria incubated with the respective mouse antisera. The nine isolates and their respective KL class denoting the capsule serotypes are indicated alongside respective histograms. **(C)** Comparison of the capsule size in bacterial isolates that show low (N = 5) *versus* high (N = 4) susceptibility to neutrophils phagocytosis. Box and whisker plots represent mean values for each group and the error bars indicate the minimum and maximum value. Mann-Whitney U-test was used for statistical analysis *p-value < 0.05, **p-value < 0.01, ns: p-value=not significant.

To further characterize the ability of AB3879 and multi strain antisera, to promote neutrophil phagocytosis of *A. baumannii* strains, we repeated the flow cytometry neutrophil phagocytosis assay with all nine Thai clinical isolates. Susceptibility to neutrophil phagocytosis when incubated in naïve mouse sera varied markedly between *A. baumannii* strains even within KL type (e.g., KL47 and KL52 strains) (**Figure 8**). Compared to control sera, the AB3879 antisera enhanced phagocytosis of the homologous AB3879 (KL10) strain to the highest degree, while multi strain sera also enhanced phagocytosis of the homologous AB3879 (KL10) and AB1615-09 (KL47) strains to the highest degree. Higher levels of phagocytosis of the heterologous AB56 (KL10) and AB1 (KL52) strains were also detected when incubated in either antisera, and for the NPRC-AB20 (KL52) strain as well in AB3879 antisera (**Figure 8A**). Overall, the neutrophil uptake data showed three broad patterns of results. The first pattern consisted of strains that were susceptible to neutrophil phagocytosis and in which antisera had little additional effect (e.g., strains AB15, AB98, and AB1492-09). The second pattern were strains that were relatively resistant to neutrophil phagocytosis in naïve sera for which addition of antisera significantly improved phagocytosis (e.g., AB1615-09, AB56, AB3879, AB1, and NPRC-AB20). Importantly, these included strains in which phagocytosis was increased by *A. baumannii* antisera that was raised against heterologous KL types (e.g., AB1 and NPRC-AB20), demonstrating a potential effect of anti-protein rather than anti-capsular antibodies. The last pattern identified was a single strain that was highly resistant to neutrophil phagocytosis under all conditions (AB55).

We observed that the *A. baumannii* capsule inhibited promotion of phagocytosis and the unencapsulated AB5075^Δwza^ strain was significantly more susceptible to neutrophil phagocytosis compared to encapsulated AB5075^WT^ strain, even in naïve sera (**Figure 7**). To further investigate the marked difference in susceptibility to neutrophil phagocytosis observed in the Thai clinical strains, we compared the bacterial cell length, as a proxy measure of capsule thickness, between the strains that showed low or no susceptibility to phagocytosis in naïve sera (AB1615-09, AB56, AB3879, NPRC-AB20 and AB55) and those strains that were highly susceptible to phagocytosis in naïve sera (AB15, AB98, AB1 and AB1492-09). No differences were observed suggesting that the capsule size between these two groups is unlikely to explain the difference in neutrophil phagocytosis (**Figure 8C**).

### Sequential Immunization Partially Protects Against Bacteremia in Mouse Models of Heterologous *A. baumannii* Infection

**Figure 9:**
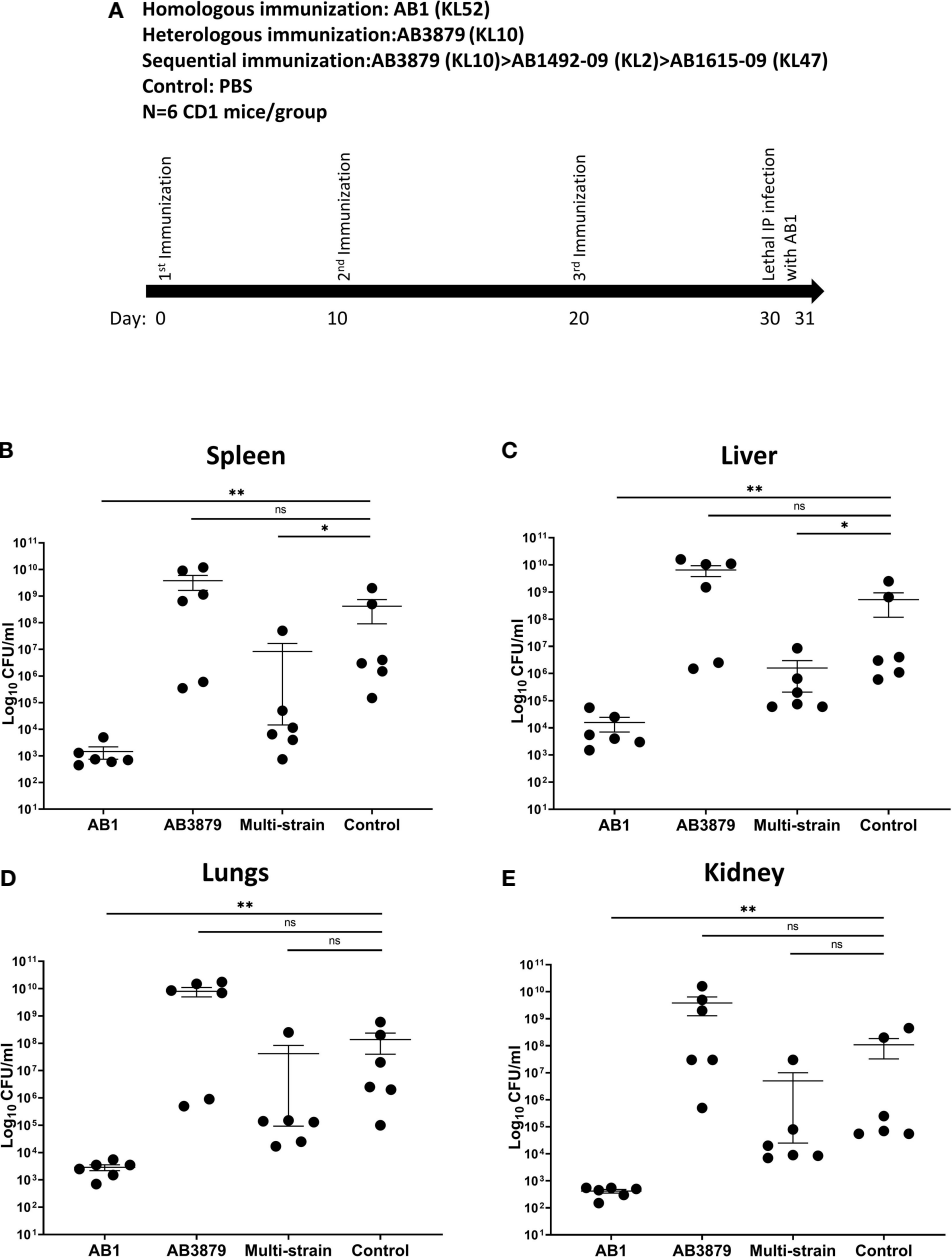
Bacterial load in the spleen, liver, lungs and kidneys from mice following lethal challenge with heterologous AB1 isolates. **(A)** CD1 mice were immunized three times with 10^6^ CFU of either AB3879, AB1, sequentially with AB3879, AB1492-09 & AB1615-09, or PBS followed by IP infection with 6.0 x 10^9^ CFU/mouse of AB1 isolate in a lethal bacteremia model of infection. The bacterial burden in the **(B)** spleen, **(C)** liver, **(D)** lungs and **(E)** kidney were determined 24 hpi. Dot plots represent individual values with lines indicating the mean and the error bars indicate standard deviations (SDs) (n = 6/group) Mann-Whitney U-test was used for statistical analysis *p-value < 0.05, **p-value < 0.01, ns: p-value=not significant.

We assessed whether the increased bacterial IgG binding and neutrophil phagocytosis observed *in vitro* with the multi strain antisera to heterologous strains, resulted in increased protection in a mouse model of *A. baumannii* bacteremia. Groups of 6 mice were immunized three times with either the single strains AB3879 (KL10) or AB1 (KL52) or sequentially with AB3879, AB1492-09 and AB1615-09 (identical to multi strain antisera group) and then challenged with the AB1 strain (**Figures 9A**). Mice immunized with the homologous strain AB1 had significantly lower bacterial CFU in the spleen, liver, lungs, and kidneys compared to control group (**Figures 9B–E**). Similar findings were observed in mice immunized with AB3879 followed by challenge with the homologous strain (**Supplementary Figure 3**), confirming homologous vaccination protected against subsequent infection.

In contrast, the bacterial burden of mice immunized with the AB3879 strain and challenged with the AB1 strain, did not differ from the control group in all organs tested (**Figures 9B–E**). However, mice immunized sequentially with three strains, all heterologous to the AB1 challenge strain, also had reduced bacterial CFU in the spleen and liver compared to control group (**Figures 9B, C**). These data demonstrate that sequential pre-exposure to heterologous *A. baumannii* strains partially protected mice from a lethal injection, indicating a protective effect of anti-protein antibodies in this serum.

## Discussion

The use of monoclonal antibodies for the treatment of multi-drug resistant bacteria is currently undergoing evaluation in clinical trials for multi-antibiotic resistant bacterial pathogens such as *Pseudomonas aeruginosa* and methicillin-resistance *Staphylococcus aureus*. These monoclonal antibodies target either the capsular polysaccharide, secreted protein toxins or surface membrane proteins such as type III secretion systems involved in host-pathogens interactions (44). Although the *A. baumannii* polysaccharide capsule is highly immunogenic and induces protective antibodies (34, 35, 45), targeting the capsule faces significant challenges. These include a wide range of described KL types (capsule serotypes), and limited knowledge of which of these, are the predominant cause of clinical cases of *A. baumannii* infections, which may also vary in different geographical locations (37, 46). For example, a total of 24 serotypes were identified among a large number of *A. baumannii* clinical isolates from Thailand (37). In contrast, a study from Vietnam found a total of 4 KL serotypes (KL2, KL49, KL58, and KL32) (46) but these together represented only 15% of the isolates in the Thai study. Similar contradictory data have been obtained using antibodies to different *A. baumannii* capsular polysaccharides with the K1 specific monoclonal antibody 13D6 recognizing 13% of the isolates tested (35), whereas C8, targeting the HUMC1 capsular polysaccharide, and anti-KL22 polyclonal sera recognized 60% of the clinical strains tested (34, 45). This variation in the proportion of strains recognized by anti-capsular antibody is likely to reflect differences between geographical sources in the predominant KL types, and perhaps for the anti-KL22 data, partial cross recognition of surface proteins found in the capsular material. These data suggest that a monoclonal therapy targeting *A. baumannii* KL types could be ineffective due to KL type diversity and would also have to be tailored to the KL types prevalent in each specific geographical region.

Developing monoclonal antibodies that recognize conserved sub-capsular protein antigens could overcome some of the challenges faced by targeting the capsular polysaccharide. Identifying which conserved proteins are potential vaccine candidates or targets of antibody therapy has been addressed using reverse vaccinology approaches for many pathogens (47). However, there are few data on acquired protective immunity to *A. baumannii* to aid in protein target antigen identification. To begin to address this, we have generated anti-*A. baumannii* sera by immunization against a single strain or sequential immunization with 3 strains, an approach that has been exploited to induce broadly reactive antibody responses in HIV (48), Influenza (49) and *Neisseria meningitidis* (50). Our results demonstrated that polyclonal mouse antisera generated following immunization with live *A. baumannii* contained a mixture of antibodies that target the polysaccharide capsule and protein targets. KL-type specific anti-capsular IgG responses predominated but using immunoblots and assays with an unencapsulated or heterologous KL type strains, we have identified additional anti-protein antigen responses. Previous data using sequential vaccination with outer membrane vesicles from three heterologous *N. meningitidis* strains improved antibody responses against genetically diverse strains and boosted the antibody titers to the highly conserved surface protein A (NspA) (50). Similarly, our data show that compared to immunization with a single strain, sequential immunization with multiple strains elicited antibodies that recognized more immunoreactive protein bands on immunoblot, boosted IgG recognition of both homologous and heterologous isolates, and resulted in enhanced phagocytosis of some heterologous isolates. Furthermore, prior immunization with three heterologous strains reduced target organ CFU in mice challenged with another *A. baumannii* strain. These data provide proof of principle that antibody to protein antigens can provide cross-protective immunity, although the ability of the capsule to inhibit antisera dependent phagocytosis remains a significant challenge.

We have used *in vitro* assays to help clarify mechanisms by which antisera might protect against *A. baumannii*. **Table 3** summarizes and allows cross-comparisons of the results from these assays. There was a lack of concordance between the whole-cell ELISA and immunoblot results, which recognized proteins in all strains, and the surface IgG binding data which showed variable reactivity to the same strains. These results suggest that the flow cytometry assay was dominated by anti-capsular antibody responses, although it clearly identified an anti-protein antigen response in some antisera/strain conditions. This was expected as the flow cytometry assay measures IgG binding to the surface of the whole bacterium and our data confirms previous data that the *A. baumannii* capsule blocks access to subcapsular protein antigens (18), whereas whole-cell ELISAs measure antibody responses to all bacterial antigens. However, there was heterologous recognition of some strains, especially in sera obtained from mice vaccinated with three different strains indicating IgG binding to heterologous *A. baumannii* strains through anti-protein antibody responses. The functional consequences of the anti-protein responses were investigated using growth inhibition in antisera and by flow cytometry neutrophil uptake assay. Inhibition of *A. baumannii* growth when cultured in broth in the presence of antisera was only caused by KL type specific anticapsular antibody. Susceptibility to neutrophil phagocytosis varied markedly between the Thai *A. baumannii* strains, an observation that needs further investigation to identify the underlying causes and whether strains that are less susceptible to neutrophil phagocytosis are more likely to cause invasive clinical infections (as has been shown for *Streptococcus pneumoniae*) (51). Although the capsule impaired both phagocytosis and antibody access to underlying protein antigens, our data showed improved phagocytosis of selected heterologous strains when incubated in sera from mice vaccinated with *A. baumannii* strains especially the multi strain antisera. The effects on neutrophil phagocytosis mirrored to an extent the IgG binding data, although with some exceptions (**Table 3**). Several studies using neutrophil depleted mice have demonstrated the important role of neutrophils in mediating resistance to both pneumonia (52) and bacteremia (53) following *A. baumannii* infection, suggesting the *in vitro* data could translate into *in vivo* protection against infection. Indeed, in mice previously immunized with the three different *A. baumannii* strains used to make the multi strain antisera, there was partial reductions in target organ CFU when infected with the heterologous AB1 strain in a bacteremia model, indicating some protective efficacy of the anti-protein antigen response. Protection is likely to be mediated by antibody (43), but this will require experimental confirmation using passive transfer of immune sera and/or B cell and T cell depletion experiments, as cell mediated immunity could also contribute to the protection observed. However, this is beyond the scope of the present work. *A. baumannii* also commonly causes pneumonia and infections on indwelling devices, urinary tract infections or soft tissue infections and additional suitable *in vivo* models will be required to comprehensively evaluate the efficacy of an antibody therapy for the treatment of all *A. baumannii* infections (54, 55). These are likely to require a degree of immunosuppression to allow *A. baumannii* to establish an infection at the target site. In summary, the *in vitro* data suggest that flow cytometry measurement of IgG binding and of neutrophil phagocytosis provide data that can correlate to *in vivo* protection against bacteremia by a heterologous strain, and hence could be used for rapid assessment of the potential protective efficacy of a particular antisera or antibody to *A. baumannii*.

**Table 3:** Summary of the *in vitro* assays measuring AB3879 and multi strain antisera effect on *A. baumannii* strains.

**Strains**			**AB3879 antisera (KL10)**				**Multi strain antisera (KL10, KL2, KL47)**			
**Unique ID**	**KL class**	**ST type**	**IgG ELISA**	**IgG FACS**	**Growth**	**PMN uptake**	**IgG ELISA**	**IgG FACS**	**Growth**	**PMN Uptake**
AB15	KL47	ST2	++	++	–	–	++++	+++	+	++
AB98	KL47	ST215	++	++++	–	-	++++	+++	+	-
AB1615-09	KL47	ST164	++	++	–	+	++++	++++	++	++++
AB56	KL10	ST2	+++	++++	+	++++	+++	++++	+	+++
AB3879	KL10	ST215	+++	+++	++	++++	+++	+++	+++	++++
AB1	KL52	ST2	++	+	–	+	+++	+++	–	++++
NPRC-AB20	KL52	ST215	++	–	–	++	++++	–	–	+
AB55	KL6	ST2	++	+	–	-	+++	+++	–	+
AB1492-09	KL2	ST2	++	+	–	+	+++	++	–	-
AB5075^WT^	KL25	ST1	++	+	–	–	++++	+++	–	++
AB5075^∆wza^	N/A		++++	+++	–	+++	++++	++++	+	++++
**Key:**				–	+	++		+++	++++	
ELISA (OD450_nm_)				<0.3	0.3-0.50	0.51-1.00		1.01-1.50	> 1.51	
FACS (MFI)				<1000	1001-10,000	10,001-50,000		50,001-500,000	>500,001	
Growth curve doubling time ratio				< 1.2	1.21-1.50	1.51-2.00		2.01- 3.0	>3.01	
PMN uptake (MFI antisera/MFI negative sera)				<1.2	1.21-1.5	1.51-2.0		2.0- 3.0	>3.01	

N/A, not applicable. Shading represents the degree of response as shown in increasing order in the key.

Successive immunization of mice with different *A. baumannii* strains may have strengthened IgG recognition of heterologous strains compared to immunization with a single strain by amplifying recall responses to conserved protein antigens. A detailed characterization of the protein targets recognized by the protective multi strain antisera is underway and will help lead to the identification of potential monoclonal antibody targets for enhancing neutrophil clearance of an *A. baumannii* infection. Although we evaluated total IgG in this study, future work will also evaluate IgG isotypes when characterizing protective antibody responses to specific *A. baumannii* proteins, to help guide the development of monoclonal antibodies that elicit specific effector functions (56). However, a key challenge to monoclonal antibody development, is the inhibitory effect of *A. baumannii* capsule to both antibody binding and neutrophil phagocytosis, as well as the genetic variation between *A. baumannii* strains which makes targeting a single antigen unlikely to protect against all strains. An effective monoclonal antibody therapy may have to target several *A. baumannii* protein antigens to help overcome these challenges.

## Data Availability Statement

The original contributions presented in the study are included in the article/**Supplementary Material**. Further inquiries can be directed to the corresponding author.

## Ethics Statement

The animal study was reviewed and approved by UCL Biological Services Ethical Committee and the UK Home Office (P64714548).

## Author Contributions

JB led the design and setup of the project. PK and PT provided bacterial strains. GK, SW, and YC performed the experiments. RS performed the bioinformatics analysis. GK, SW, YC, and CK analyzed the data. GK and JB wrote the manuscript. All authors contributed to the article and approved the submitted version.

## Funding

This work was undertaken at UCLH/UCL who received a proportion of funding from the Department of Health’s NIHR Biomedical Research Centre’s funding scheme and was also supported by an MRC DPFS MR/S004394/1 to RS, BW, GL and JB. The funders had no role in study design, data collection and interpretation, or the decision to submit the work for publication.

## Conflict of Interest

The authors declare that the research was conducted in the absence of any commercial or financial relationships that could be construed as a potential conflict of interest.

## Publisher’s Note

All claims expressed in this article are solely those of the authors and do not necessarily represent those of their affiliated organizations, or those of the publisher, the editors and the reviewers. Any product that may be evaluated in this article, or claim that may be made by its manufacturer, is not guaranteed or endorsed by the publisher.

## Supplementary Material

Supplementary Figure 1 Growth inhibitory activity of unencapsulated AB5075^Δwza^ isolates by mouse antisera. 10^2^ CFU of un-encapsulated AB5075^Δwza^ strain was incubated in triplicate with either, AB5075^WT^ antisera (black line, solid diamond), AB5075^Δwza^ antisera (black line, open diamond) and sera from non-immunized mice (grey line, solid circle) **(A)** or AB3879 antisera (black line, solid square), multi strain antisera (black line, open square), and sera from non-immunized mice (grey line, solid circle) **(B)** and the OD_600nm_ measured every 30 minutes over 24 hours.

Supplementary Figure 2 Growth inhibitory activity on a panel of four *A. baumannii* isolates by mouse antisera. 10^2^ CFU of a panel of *A. baumannii* isolates were incubated in triplicate with either AB3879 antisera (left panel) or multi strain antisera (right panel) and the OD_600nm_ measured every 30 minutes for 24 hours. Sera from non-immunized mice was included as a control (open symbols). Solid symbols represent respective anti-*A. baumannii* antisera. Data from two independent experiments is shown. The five isolates are grouped by their capsule serotype and indicated at the top of each graph in brackets. Round, square and triangle symbols represent ST2, ST215, and ST164 MSLT types respectively.

Supplementary Figure 3 Bacterial load in the spleen, liver and lungs from mice following lethal challenge with homologous AB3879 strain. CD1 mice were immunized three times with 10^6^ CFU of the AB3879 or PBS followed by IP infection with 2.0 x 10^7^ CFU/mouse of the homologous AB3879 strain in a bacteremia model of infection. The bacterial burden in the spleen, liver and lungs were determined 24 hpi. Dot plots represent individual values with lines indicating the mean and the error bars indicate standard deviations (SDs) (n=15/group) Mann-Whitney U-test was used for statistical analysis *p-value < 0.05, **p-value< 0.001, ***p-value < 0.0001, ns: p-value= not significant.
