# Supplementary figures and images for: Sequential Vaccination With Heterologous Acinetobacter baumannii Strains Induces Broadly Reactive Antibody Responses
Source: Front Immunol. 2021 Jul 30;12:705533. doi: 10.3389/fimmu.2021.705533 (PMC8363311; doi:10.3389/fimmu.2021.705533)

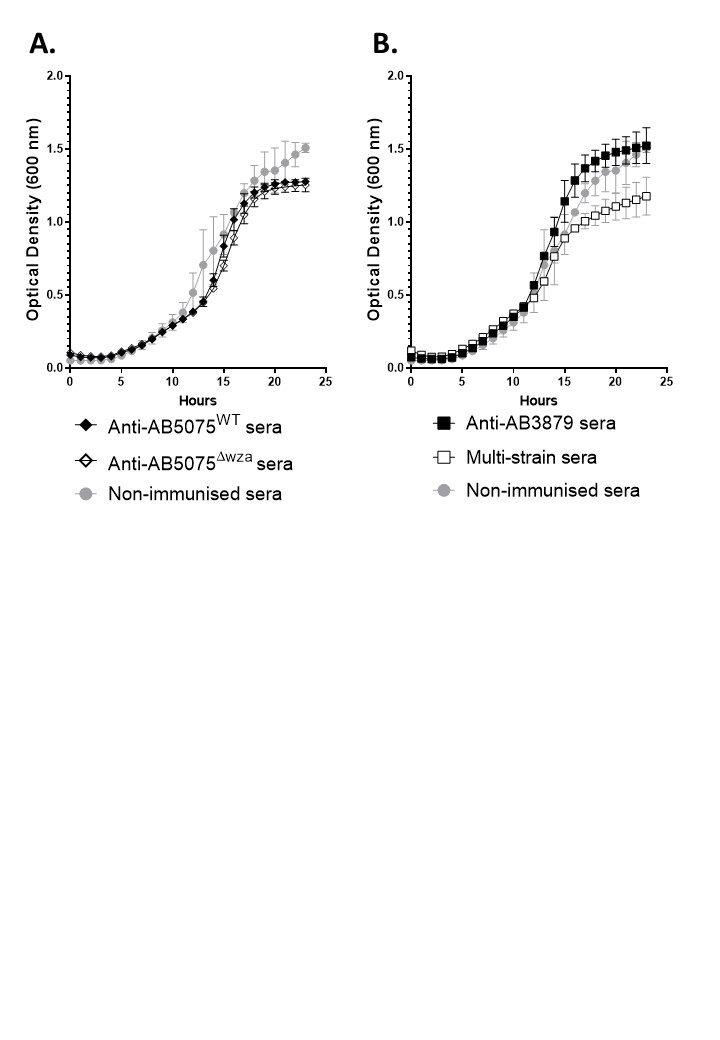

Supplement: Supplementary Figure 1 — Growth inhibitory activity of unencapsulated AB5075Δwza isolates by mouse antisera. 102 CFU of un-encapsulated AB5075Δwza strain was incubated in triplicate with either, AB5075WT antisera (black line, solid diamond), AB5075Δwza antisera (black line, open diamond) and sera from non-immunized mice (grey line, solid circle) (A) or AB3879 antisera (black line, solid square), multi strain antisera (black line, open square), and sera from non-immunized mice (grey line, solid circle) (B) and the OD600nm measured every 30 minutes over 24 hours. [file Image_1.jpeg]

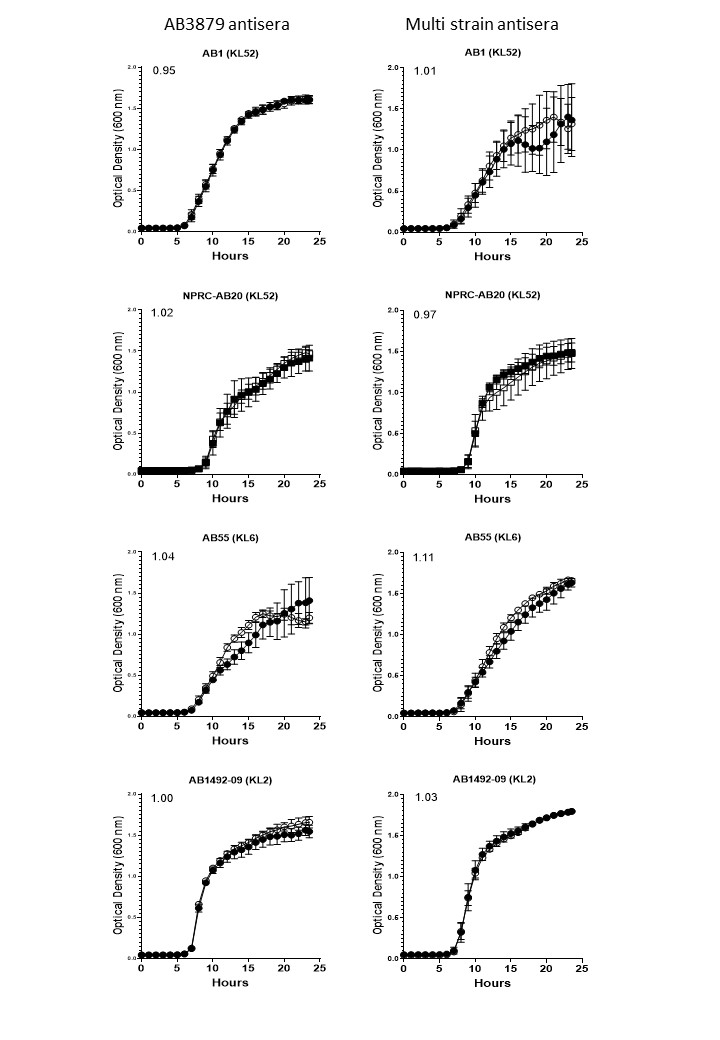

Supplement: Supplementary Figure 2 — Growth inhibitory activity on a panel of four A. baumannii isolates by mouse antisera. 102 CFU of a panel of A. baumannii isolates were incubated in triplicate with either AB3879 antisera (left panel) or multi strain antisera (right panel) and the OD600nm measured every 30 minutes for 24 hours. Sera from non-immunized mice was included as a control (open symbols). Solid symbols represent respective anti-A. baumannii antisera. Data from two independent experiments is shown. The five isolates are grouped by their capsule serotype and indicated at the top of each graph in brackets. Round, square and triangle symbols represent ST2, ST215, and ST164 MSLT types respectively. [file Image_2.jpeg]

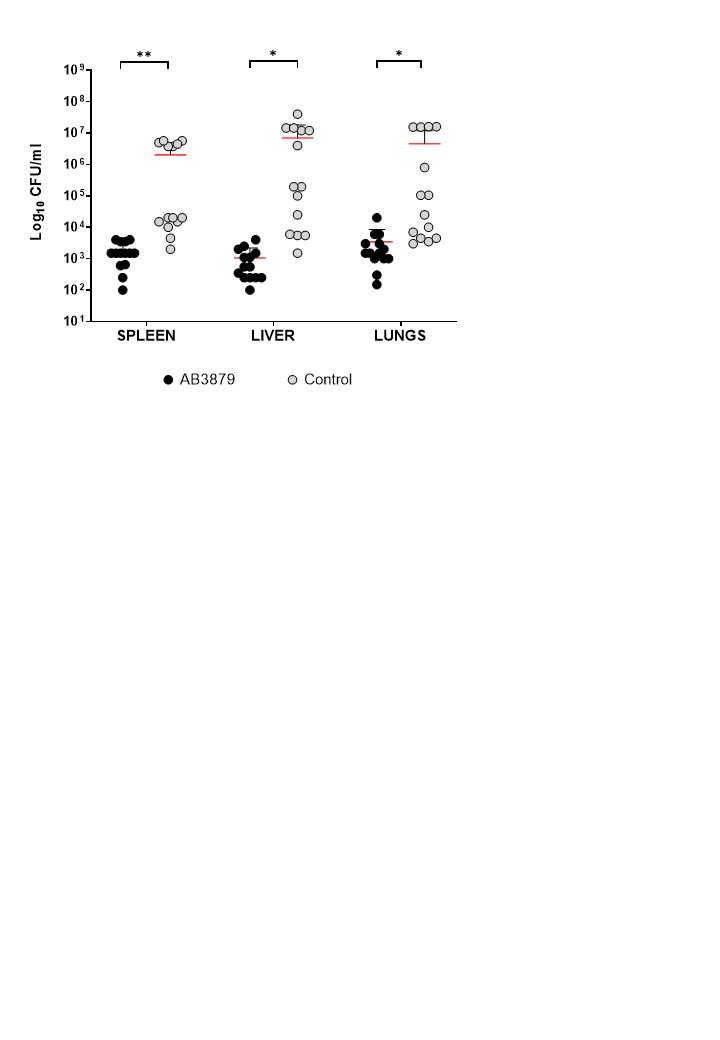

Supplement: Supplementary Figure 3 — Bacterial load in the spleen, liver and lungs from mice following lethal challenge with homologous AB3879 strain. CD1 mice were immunized three times with 106 CFU of the AB3879 or PBS followed by IP infection with 2.0 x 10^7^ CFU/mouse of the homologous AB3879 strain in a bacteremia model of infection. The bacterial burden in the spleen, liver and lungs were determined 24 hpi. Dot plots represent individual values with lines indicating the mean and the error bars indicate standard deviations (SDs) (n=15/group) Mann-Whitney U-test was used for statistical analysis *p-value < 0.05, **p-value< 0.001, ***p-value < 0.0001, ns: p-value= not significant. [file Image_3.jpeg]
